# A Geomagnetic Estimate of Heliospheric Modulation Potential over the Last 175 Years

**DOI:** 10.1007/s11207-024-02316-9

**Published:** 2024-06-19

**Authors:** Mathew J. Owens, Luke A. Barnard, Raimund Muscheler, Konstantin Herbst, Mike Lockwood, Ilya Usoskin, Eleanna Asvestari

**Affiliations:** 1 Department of Meteorology, University of Reading, Earley Gate, PO Box 243, Reading, RG6 6BB UK; 2 Lund University, Box 117, SE-221 00 Lund, Sweden; 3 Christian-Albrechts-Universität zu Kiel, Christian-Albrechts-Platz 4, 24118 Kiel, Germany; 4 Space Physics and Astronomy Research Unit and Sodankyla Geophysical Observatory, University of Oulu, 90014 Oulu, Finland; 5 Department of Physics, University of Helsinki, Helsinki, Finland

## Abstract

Galactic cosmic rays (GCRs) interact with the Earth’s atmosphere to produce energetic neutrons and cosmogenic radionuclides, such as ^14^C. The atmosphere is partially shielded from the interstellar GCR spectrum by both the geomagnetic and solar magnetic fields. Solar shielding is often expressed as the heliospheric modulation potential $\phi $, which consolidates information about the strength and structure of the solar magnetic field into a single parameter. For the period 1951 to today, $\phi $ can be estimated from ground-based neutron monitor data. Prior to 1950, ^14^C in tree rings can be used to estimate $\phi $ and hence the solar magnetic field, back centuries or millennia. Bridging the gap in the $\phi $ record is therefore of vital importance for long-term solar reconstructions. One method is to model $\phi $ using the sunspot number (SN) record. However, the SN record is only an indirect measure of the Sun’s magnetic field, introducing uncertainty, and the record suffers from calibration issues. Here we present a new reconstruction of $\phi $ based on geomagnetic data, which spans both the entire duration of the neutron monitor record and stretches back to 1845, providing a significant overlap with the ^14^C data. We first modify and test the existing model of $\phi $ based on a number of heliospheric parameters, namely the open solar flux $F_{S}$, the heliospheric current sheet tilt angle $\alpha $, and the global heliospheric magnetic polarity $p$. This modified model is applied to recently updated geomagnetic estimates of $F_{S}$ and cyclic variations of $\alpha $ and $p$. This approach is shown to produce an annual estimate of $\phi $ in excellent agreement with that obtained from neutron monitors over 1951 – 2023. It also suggests that ionisation chamber estimates of $\phi $ – which have previously been used to extend the instrumental estimate back from 1951 to 1933 – are not well calibrated. Comparison of the new geomagnetic $\phi $ with ^14^C estimates of $\phi $ suggests that the long-term trend is overestimated in the most recent ^14^C data, possibly due to hemispheric differences in the Suess effect, related to the release of carbon by the burning of fossil fuels. We suggest that the new geomagnetic estimate of $\phi $ will provide an improved basis for future calibration of long-term solar activity reconstructions.

**Supplementary Information:**

The online version contains supplementary material available at 10.1007/s11207-024-02316-9.

## Introduction

Solar magnetic activity waxes and wanes with the approximately 11-year solar cycle (e.g. Vennerstrom et al., 2016; Kilpua, Koskinen, and Pulkkinen, 2017; Owens et al., 2021; Hathaway, 2010). However, the amplitudes of solar cycles are also modulated on centennial and millennial time scales, resulting in longer-term variations in the solar activity, referred to as “space climate” (Usoskin, 2023). Direct observations of the solar magnetic field, such as the approximately 60-year record of near-Earth solar wind conditions from in situ spacecraft observations (King and Papitashvili, 2005) are generally too short to draw meaningful conclusions about space climate. Thus indirect proxies must be used. The sunspot observations, which are available with varying coverage back approximately 400 years, are the most widely used proxy (Vaquero et al., 2016; Clette et al., 2023). These data are an invaluable resource, especially considering that 400 years likely spans the full range of solar variability exhibited over recent millennia (Acero et al., 2018). However, the intercalibration of sunspot observers, particularly during a dearth of observations in the 18th century (Hayakawa et al., 2022), makes it challenging to construct a homogeneous sunspot number (SN) record over the full 400-year interval. However, incomplete records do exist back to 1610 (e.g. Arlt, 2011; Usoskin et al., 2015). Furthermore, even putting the calibration issues aside, SN is only a visible proxy for solar magnetic activity, rather than a direct measure. Although many physical measures of solar magnetic activity do correlate well with SN over the limited parameter range for which simultaneous observations are available, there is high uncertainty in extrapolating outside this parameter space, particularly to very low solar activity levels such as Maunder minimum-like conditions (e.g. Usoskin et al., 2015).

The longest reconstructions of space climate are obtained from records of radioactive isotopes created by galactic cosmic rays (GCRs) interacting with Earth’s atmosphere. These cosmogenic radionuclides, such as ^14^C and ^10^Be, are stored in natural, independently dateable archives, such as tree rings and ice sheets, respectively (Lal and Peters, 1967; Beer et al., 2013; Usoskin, 2023). Interpretation of these data is not straightforward. For ^14^C, the terrestrial carbon cycle (and hence Earth’s climate system) must be modeled (e.g. Stuiver and Quay, 1980; Roth and Joos, 2013). For ^10^Be, local climate factors can be important, meaning that multiple ice cores from different geographic locations must be used (e.g. Muscheler et al., 2007; Zheng et al., 2023; Golubenko et al., 2022). Accounting for these factors allows the radionuclide production rate to be estimated, from which we can infer the GCR flux at the top of the atmosphere. Assuming a constant interstellar GCR spectrum, the combined geomagnetic and heliospheric shielding effect can be computed as a function of time (Kovaltsov, Mishev, and Usoskin, 2012; Herbst, Muscheler, and Heber, 2017). There are two approaches to separating these two contributions. The first is to use geomagnetic magnetic field reconstructions (Alken et al., 2021) to isolate the effect of heliospheric shielding (Vonmoos, Beer, and Muscheler, 2006; Kovaltsov and Usoskin, 2010). The second is to separate different frequencies of geomagnetic and heliospheric shielding (Berggren et al., 2009; Nguyen, 2023). However, there is debate about the degree to which both geomagnetic and solar modulation factors compete with each other on the (multi)centennial timescale (e.g. Snowball and Muscheler, 2007; Usoskin, 2017; Pavón-Carrasco et al., 2018) and thus how effectively they can be disentangled by simple filtering.

Heliospheric shielding of GCRs results from constraints on their effective transport through the heliosphere to Earth’s atmosphere, which is itself the result of a complex set of physical processes, at a range of temporal and spatial scales (Parker, 1965). These can be collectively described in terms of diffusion of GCRs along and perpendicular to the heliospheric magnetic field (HMF), outward advection of GCRs by the solar wind, adiabatic deceleration of GCRs owing to a stronger HMF close to the Sun, and gradient and curvature drifts of GCRs in the global HMF. Although three-dimensional numerical models exist that solve for GCR transport accounting for these processes (Potgieter, 2013; Moloto and Engelbrecht, 2020), the highly structured nature of the turbulent solar wind and HMF means that simplifying parameterizations are required, particularly for the diffusion tensor. For these reasons, it is often convenient to instead use the one-dimensional “force-field” approximation (Gleeson and Axford, 1968; Usoskin et al., 2005; Herbst et al., 2010; Moraal, 2013; Caballero-Lopez, Engelbrecht, and Richardson, 2019). In this limit, the mean energy loss per GCR particle in the heliosphere can be described by a single parameter, referred to as the heliospheric modulation potential $\phi $.

**Figure 1:**
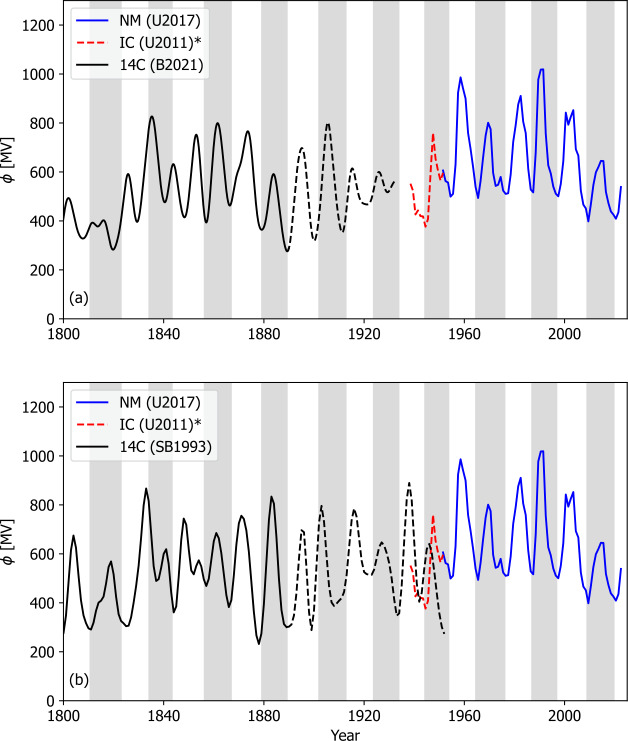
Bridging the gap between ^14^C and instrumental heliospheric modulation potential, $\phi$, estimates. All time series are annual resolution. Grey- and white-shaded regions show even- and odd-numbered solar cycles, respectively (from minimum to minimum). The neutron-monitor (NM)-based estimate of $\phi$ from U2017 is shown in blue. The uncertain Usoskin, Bazilevskaya, and Kovaltsov (2011) extension based on ionisation chamber (IC) data is shown as a red dashed line. Black lines show the ^14^C-based $\phi$ estimates. Panel (a) shows the Brehm et al. (2021) estimate. Panel (b) shows the $\phi$ estimate from the University of Washington ^14^C record (Stuiver and Braziunas, 1993). In both panels, the dashed lines indicate the approximate period of increased uncertainty in ^14^C $\phi$ due to dilution of natural ^14^C in the atmosphere due to burning of fossil fuels. A gap exists between the reliable instrumental-based estimates (NM and/or IC) and ^14^C estimates of $\phi$.

Annual measurements of ^10^Be have been available for some time (e.g. Beer et al., 1990; Muscheler et al., 2016). However, local climate effects make reconstruction of the solar signal – and hence $\phi $ – somewhat challenging, at least at annual resolution (Zheng et al., 2021; Paleari et al., 2023). Recent developments in ^14^C analysis have enabled $\phi $ reconstructions at the annual timescale (Stuiver and Braziunas, 1993) over the last millennium (Brehm et al., 2021). To test and calibrate these $\phi $ reconstructions, it is desirable to be able to compare them with modern, more direct observations. Ground-based neutron monitor records provide precise, high-resolution data on GCR intensity back to 1951 (e.g. Väisänen, Usoskin, and Mursula, 2021). Indeed, these data provide a basis for the most direct, multidecadal estimate of heliospheric modulation potential, which we here refer to as $\phi _{NM}$. Figure 1 shows $\phi _{NM}$ from Usoskin et al. (2017), based on the interstellar GCR spectrum estimated by Vos and Potgieter (2015). This series is referred to as U2017.

Unfortunately, due to atmospheric nuclear testing, radiocarbon-based estimates of $\phi $ are not possible after around 1950, with the Brehm’s et al. (2021) ^14^C series ending in 1933. This is shown as the black line in Figure 1a. The $\phi $ estimate based on an earlier (but shorter) annual ^14^C record from the University of Washington (Stuiver and Braziunas, 1993) is shown in Figure 1b. The extensive use of fossil fuels after the industrial revolution led to the dilution of the natural ^14^C in the atmosphere – this is known as the “Suess effect”. Carbon cycle models account for this effect, but hemispheric differences and lack of overlap with $\phi _{NM}$ introduce uncertainty. While there is no “switch on” date for the Suess effect, it is known to be strong after 1890 (Baxter and Walton, 1970), which is approximately illustrated by the dashed black lines in Figure 1.

It is highly desirable to extend $\phi _{NM}$ prior to 1951 to provide overlap with the radionuclide estimates. Ground-based ionisation chamber (IC) data provide a possibility to extend this dataset back to 1931 (McCracken and Beer, 2007), though careful calibration is required. Usoskin, Bazilevskaya, and Kovaltsov (2011) attempted to homogenise the NM and IC data, though cautioned that there were numerous caveats and large uncertainties in the resulting NM-IC $\phi $ record. Even with the IC extension (red dashed line in Figure 1), there remains limited overlap between the time intervals reliably covered by instrumental data and annual ^14^C reconstructions.

It is highly desirable to be able to more completely bridge this gap with a reliable $\phi $ estimate. Alanko-Huotari et al. (2007) and Asvestari and Usoskin (2016), hereafter AU2016, outlined a model of $\phi $ based upon a number of observable heliospheric parameters. These were the open solar magnetic flux $F_{S}$, the heliospheric current sheet tilt angle $\alpha $, and the global solar magnetic polarity $p$. The angle $\alpha $ is found to vary quasi-periodically with solar cycle phase, whereas $p$ alternates between −1 and +1 at the maximum of each solar cycle. Thus only an observational estimate of $F_{S}$ and knowledge of the solar cycle start/end dates are required to produce a $\phi $ reconstruction. AU2016 used a sunspot-based estimate of $F_{S}$ (Lockwood, Owens, and Barnard, 2014; Lockwood and Owens, 2014) for this purpose. Although this potentially allows $\phi $ to be estimated back to 1610, there are two issues with this approach. Firstly, the conversion from SN to $F_{S}$ is based on a semi-empirical model (Owens and Lockwood, 2012; Krivova et al., 2021), which introduces uncertainty into $\phi $ estimate. Secondly, the SN-based $F_{S}$ estimate is only as good as the underlying SN record, which is known to be problematic prior to around 1750 and possibly even before 1850 (Hayakawa et al., 2022; Clette et al., 2023). In addition, not using the amplitude of sunspot number cycles in its derivation has the advantage of giving a value of $\phi $ that can be used to check sunspot number calibrations. We note that Usoskin et al. (2021) used the $F_{S}$ to represent the solar activity variability over the past millennium.

We here take a different approach to bridging the gap between radionuclides and estimates of $\phi $ based on instrumental data. A recent geomagnetic $F_{S}$ reconstruction is used in place of the SN-based estimate. While this only enables $\phi $ to be estimated back to 1845, previous work has shown the geomagnetic $F_{S}$ is far more accurate than the SN equivalent; in particular, they do not rely on methods that could introduce long-term drifts (Owens et al., 2016). We additionally reformulate the AU2016 model to use a simpler combination of the same component heliospheric parameters. Combined, these developments provide the most accurate estimate to date of $\phi $ prior to 1951, which will enable better calibration of the ^14^C- and ^10^Be-based reconstructions in the future.

## Data

**Figure 2:**
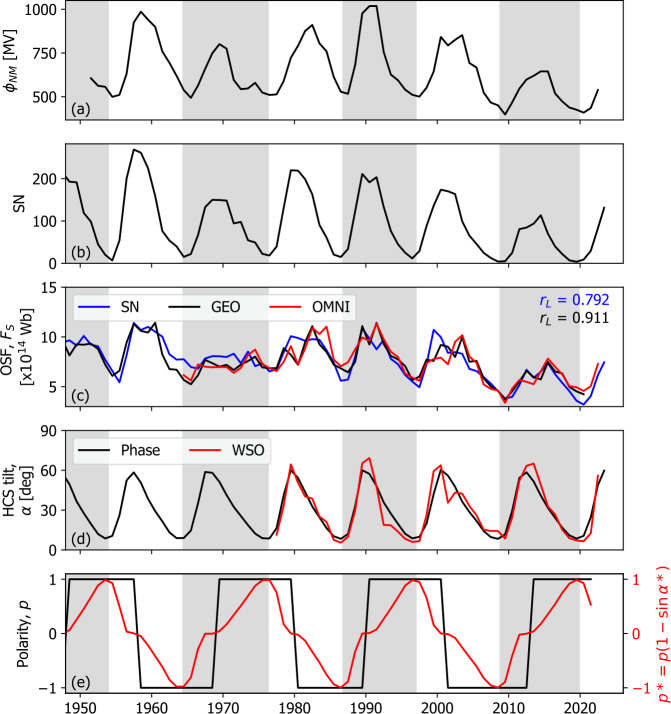
The annual time series used in this study. Grey- and white-shaded panels show even- and odd-numbered solar cycles, respectively. (**a**) The heliospheric modulation potential reconstructed from neutron monitor observations $\phi _{NM}$ by U2017. (**b**) Sunspot number SN. (**c**) Open solar flux $F_{S}$ from (red) the OMNI near-Earth in situ solar wind observations, (black) reconstructed from geomagnetic observations, and (blue) reconstructed from SN. (**d**) The heliospheric current sheet tilt angle $\alpha $ from (red) potential-field source-surface models of WSO photospheric magnetic field observations and (black) assuming an average variation of $\alpha $ with solar cycle phase. (**e**) Global solar polarity $p$ (black) and the effective polarity parameter $p*$ (red).

Figure 2 summarises the main data used in this study, all at annual resolution. Figure 2a shows the neutron monitor-based estimate of the heliospheric modulation potential $\phi _{NM}$ from U2017, based on the local interstellar spectrum parameterization by Vos and Potgieter (2015). This series, further denoted as U2017, is the $\phi $ that we attempt to reconstruct on the basis of the other solar and heliospheric series in the lower panels. A version of this reconstruction was recently produced at higher resolution (Väisänen et al., 2023), but it is nearly identical to U2017 at annual resolution.

Figure 2b shows the Sunspot Index and Long-term Solar Observations Version 2.0 (SILSOv2) sunspot number (SN) record (Clette and Lefèvre, 2016; Clette et al., 2023). While not directly used in the reconstruction of $\phi $, this series is pertinent to the present study as we require an estimate of the solar-cycle phase and hence the start/end dates of individual cycles. To provide unambiguous estimates of the start date, we define the start of the cycle using the time of the sharp increase in average sunspot latitude (Owens et al., 2011, 2024). The resulting solar cycles are shown as white- and grey-shaded regions in Figure 2. The timing of new cycles corresponds closely with the minima in SN, as expected. Solar-cycle phase is then taken to vary linearly between 0 and 1 between cycle start and end times.

The open solar flux $F_{S}$ is shown in Figure 2c. The red line shows the best estimate from in situ spacecraft observations in near-Earth space using the OMNI dataset (King and Papitashvili, 2005). This is computed by taking 20-hour averages of the radial magnetic field component $B_{R}$ and then computing $F_{S} = 4 \pi r^{2} |B_{R}|$, where $r$ is 1 AU. This has been shown to approximate the “strahl-based” method, which uses suprathermal electron observations to identify locally inverted $B_{R}$, which do not map directly back to the Sun (Owens et al., 2017; Frost et al., 2022). There is a clear solar cycle trend in $F_{S}$, as well as considerable variation from cycle to cycle. The black line shows $F_{S}$ computed from geomagnetic data. Specifically, it uses the magnetic field intensity $B$ and solar wind speed $V$, reconstructions from Lockwood et al. (2022), and produces an $F_{S}$ estimate using Parker spiral theory (Parker, 1958) and calibration against the OMNI series (Owens et al., 2024; Lockwood and Owens, 2024). The linear correlation coefficient $r_{L}$ between them is 0.91. The blue line shows the best SN-based estimate of $F_{S}$ (Owens et al., 2024), which has $r_{L} = 0.79$ with the OMNI estimate. We find a very similar value using the Krivova et al. (2021) SN-based estimate of $F_{S}$. Using a Meng-Z test, this is significantly lower than the geomagnetic $F_{S}$ correlation at the 99% confidence level.

**Figure 3:**
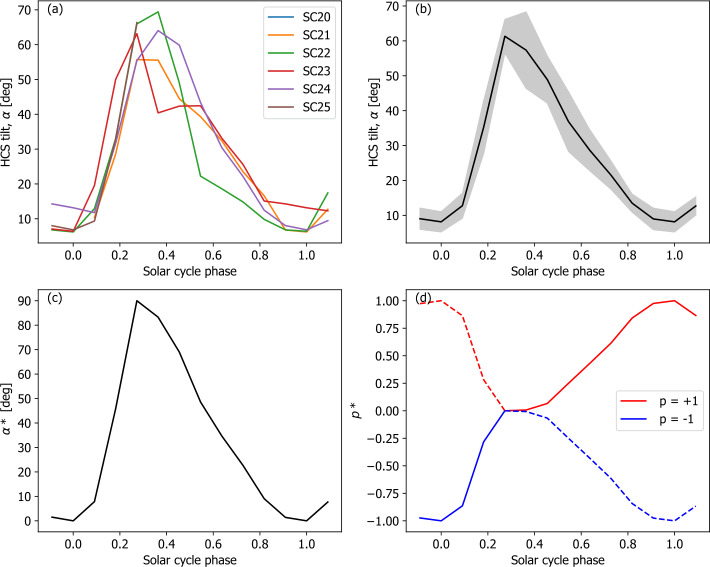
Average solar-cycle variations in properties used to estimate $\phi $. (**a**) Heliospheric current sheet (HCS) tilt angle $\alpha $ as a function of solar cycle phase. $\alpha $ is estimated using potential-field source-surface solutions to WSO magnetograms. (**b**) The averaged solar-cycle variation of $\alpha $ (black) with one standard deviation (grey). (**c**) $\alpha *$, the cycle-averaged variation $\alpha $ scaled to vary between 0 and 90^∘^. (**d**) The effective polarity $p* = p (1 - \sin \alpha *)$ for $p=+1$ (red) and $p=-1$ (blue). The solid lines show the average variation for an even-numbered solar cycle (i.e. a transition from $p=-1$ to $p=+1$), whereas the dashed lines show an odd-numbered solar cycle.

Heliospheric current sheet (HCS) tilt angle $\alpha $ estimates are obtained from the Wilcox Solar Observartory (WSO) photospheric magnetograms using a potential-field source-surface model (Hoeksema, 1991). Although there is uncertainty in HCS tilt introduced by both the input magnetograms and the potential-field modelling, there is good agreement with the available in situ observations at the annual time scale (e.g. see Figure 13 in Owens and Forsyth, 2013). The WSO-based estimates of $\alpha $ are available back to 1977 and are shown in red in Figure 2d. It has been noted that $\alpha $ is very repeatable from cycle to cycle (Alanko-Huotari et al., 2007; Owens, Crooker, and Lockwood, 2011). We exploit this fact to reconstruct $\alpha $ prior to the availability of WSO data. Figure 3a shows $\alpha $ as a function of the solar-cycle phase, which effectively normalises for variable solar-cycle length. A trend for the HCS remaining higher in the declining phase (i.e. solar phase between 0.5 and 0.8) during odd cycles than during even cycles, which was identified for Solar Cycles (SCs) 21 through 23 (Cliver and Ling, 2001), does not appear to hold for SC24. Thus all cycles are combined into a single variation in this study. Figure 3b shows the resulting average solar-cycle variation, with the standard deviation as the shaded area. This average HCS variation is shown as a black line in Figure 2d and values recorded in Table 1. The major deviation of the average profile to the original observation is the peak around 1990 and the subsequent declining phase variation.

**Table 1:** The average solar-cycle variation of the heliospheric current sheet tilt angle ($\alpha $), the scaled HCS tilt angle ($\alpha *$) and the effective polarity ($p*$) for $p=+1$ and $p=-1$.

Solar cycle phase	HCS tilt, *α* [deg]	*α*∗ [deg]	Effective polarity, *p*∗	
			*p* = +1	*p* = −1
−0.09	9.0	1.5	0.97	−0.97
0.00	8.1	0.0	1.00	−1.00
0.09	12.8	7.8	0.86	−0.86
0.18	35.2	45.9	0.28	−0.28
0.27	61.3	90.0	0.00	−0.00
0.36	57.4	83.3	0.01	−0.01
0.45	48.9	69.0	0.07	−0.07
0.55	36.9	48.6	0.25	−0.25
0.64	28.7	34.7	0.43	−0.43
0.73	21.5	22.7	0.61	−0.61
0.82	13.5	9.1	0.84	−0.84
0.91	9.0	1.4	0.98	−0.98
1.00	8.1	0.0	1.00	−1.00
1.09	12.7	7.7	0.87	−0.87

Finally, to compute $\phi $, we require an estimate of the heliospheric polarity $p$. This property is taken to be +1 when the northern pole of the Sun is primarily positive in magnetic flux and −1 when it is primarily negative. The polar reversal times can be estimated directly from photospheric magnetic field observations and are found, on average, to occur at a solar-cycle phase of 0.35 (Thomas, Owens, and Lockwood, 2013). Using this transition time, the resulting $p$ time series is shown in black in Figure 2e. By definition there is a discontinuous change in $p$ at the time of the polarity reversal. However, the heliospheric modulation of GCRs is not expected to behave in such a manner. In fact, the heliospheric polarity is only really a meaningful concept when the large-scale solar magnetic dipole is approximately rotation-aligned (i.e. when $\alpha $ is low, around solar minimum). Considering the extreme case, when $\alpha $ is approximately $90^{\circ}$, $p$ is effectively undefined and is expected to have no net effect on GCR modulation. To account for this, we define the effective polarity

1 $$ p* = p (1 - \sin \alpha *), $$

where $\alpha *$ is the HCS tilt angle $\alpha $ scaled to reach a minimum value of $0^{\circ}$ and a maximum value of $90^{\circ}$ each cycle:

2 $$ \alpha * = \frac{\pi}{2} \left ( \frac{\alpha - \alpha _{MIN}}{\alpha _{MAX} - \alpha _{MIN}} \right ). $$

For the cycle-averaged variation in $\alpha $ from WSO observations, we find $\alpha _{MIN} = 8^{\circ}$ and $\alpha _{MAX} = 61^{\circ}$. This variation is shown in Figure 2c. Thus, like $p$, $p*$ reaches a maximum/minimum value of +1/−1 at solar minimum. But $p*$ instead varies continuously and is zero at the time of maximum HCS inclination (i.e. solar maximum). This is shown in Figure 2d, and values are given in Table 1.

## Modelling $\phi $ Using OMNI $F_{S}$

The flux of GCRs at a location in the heliosphere is modulated via the scattering of GCRs by structures (or “scattering inhomogeneities”) in the heliospheric magnetic field, convecting outwards with the solar wind. Thus $\phi $ is expected to be closely related to the global magnetic field strength and structure. Indeed, GCR propagation is often modelled as a diffusion process where the diffusion coefficient is a function of the HMF strength (Li, Beacom, and Peter, 2022).

There are a number of measurable factors that can act as proxies for GCR modulation and thus from which $\phi $ can be approximated. To first order, the ability of the global HMF to modulate GCRs is best characterised by $F_{S}$, the total unsigned magnetic flux threading the solar source surface. There is no reason, however, to expect modulation to vary linearly with $F_{S}$. Second-order effects then need to be considered. Structure within the HMF, such as scattering inhomogeneities, is strongly ordered by the location of the heliospheric current sheet (HCS) (e.g. Owens, 2020). When the HCS is confined to the solar equator, such as close to solar minimum, GCR scattering is expected to be lower than that when the HCS extends to all latitudes, such as at solar maximum (Smith, 1990). Finally, the global polarity of the solar field is known to affect GCR propagation through changing large-scale drift patterns (Jokipii, Levy, and Hubbard, 1977).

AU2016 combined these properties in the following form:

3 $$ \phi _{E2016} [MV] = \phi _{0} {F_{S}} ^{n - \alpha /\alpha _{0}} (1 - \beta p), $$

where $F_{S}$ is the open solar flux in units of $\times 10^{14}$ Wb (however, see discussion below), $\alpha $ is the HCS tilt angle in degrees, and $p$ is the polarity (either +1 or −1). The parameters $\phi _{0}$, $n$, $\alpha _{0}$, and $\beta $ are free to be empirically determined by fitting to a known $\phi $ estimate, typically from neutron monitors (e.g. Usoskin, Bazilevskaya, and Kovaltsov, 2011). AU2016 applied the equation to a sunspot-based estimate of $F_{S}$ and an average $\alpha $ based on WSO observations up to 2013. They determined $\phi _{0} = 1474$ MV, $\alpha _{0} = 150^{\circ}$, n = 1.03 and $\beta = 0.095$. Using this model, they reported a linear correlation coefficient $r_{L}$ of 0.88 and showed a good level of agreement between the model and observed $\phi $ time series.

We here re-compute these coefficients for the latest revised and updated $F_{S}$ and $\alpha $ values and fit the model to the U2017 estimate of $\phi _{NM}$. However, in order to proceed, it is necessary to note that the $F_{S}$ values used in AU2016 must actually have been in units of $10^{15}$ Wb, not $10^{14}$ Wb as stated (this would be consistent with the $F_{S}$ magnitudes shown in their Figure 5). To be consistent with AU2016, we here obtain parameters for $F_{S}$ in units of $10^{15}$ Wb, the reason being that a change of units from $10^{14}$ Wb to $10^{15}$ Wb does not simply result in a change of $\phi _{0}$; the exponent of $F_{S}$ is dependent on the variable $\alpha $ and the constant $\alpha _{0}$, and thus the waveform of the resulting $\phi $ time series is different for different units of $F_{S}$. It is partly for this reason that we below propose simpler relations between $\phi $ and $F_{S}$, where this is not the case.

Using the OMNI estimates of $F_{S}$ (in units of $10^{15}$ Wb) and the observed WSO $\alpha $ series (in degrees), with the polarity reversal assumed to occur at a solar cycle phase of 0.35, we find best-fit parameters $\phi _{0} = 827$ MV, $n = 1.02$, $\alpha _{0} = 119^{\circ}$, and $\beta = 0.0166$. These values are also recorded in Table 2. As $\alpha $ has a typical value of $30^{\circ}$, this means that the AU2016 model is – to first order – scaling as $\phi \sim F_{S} ^{0.77}$. Owing to the different functional forms, it is difficult to interpret $\alpha _{0}$ and $\beta $ in terms of the relative contributions of $\alpha $ and $p$ to $\phi $. Note that $\beta = 0.0166$ and $p = \pm 1$ mean that the polarity variation is modulating $\phi $ by around 3%, i.e. the polarity is not a significant contribution to $\phi $ in this model.

**Table 2:** A table of $\phi $ model best-fit parameters for four sets of input heliospheric variables.

Inputs	Overlap	Asvestari 2016						Updated model, O2024					
		$\phi _{0}$ [MV]	*n*	$\alpha _{0}$ [deg]	*β*	$r_{L}$	MAE	$\phi _{0}$ [MV]	*n*	*A*	*B*	$r_{L}$	MAE
OMNI $F_{S}$ WSO *α*	1977 – 2021	827	1.02	119	0.0166	0.891	59.7	642	0.665	0.488	−0.0319	0.940	44.8
OMNI $F_{S}$ Average *α*	1964 – 2021	814	1.09	85	0.0151	0.865	64.3	601	0.636	0.622	−0.0563	0.934	45.6
GEO $F_{S}$ Average *α*	1951 – 2021	835	1.05	123	0.0458	0.891	55.6	674	0.685	0.382	−0.0987	0.926	44.7
SN $F_{S}$ Average *α*	1951 – 2021	810	0.911	162	0.0399	0.853	65.0	647	0.594	0.404	−0.0742	0.890	54.8

**Figure 4:**
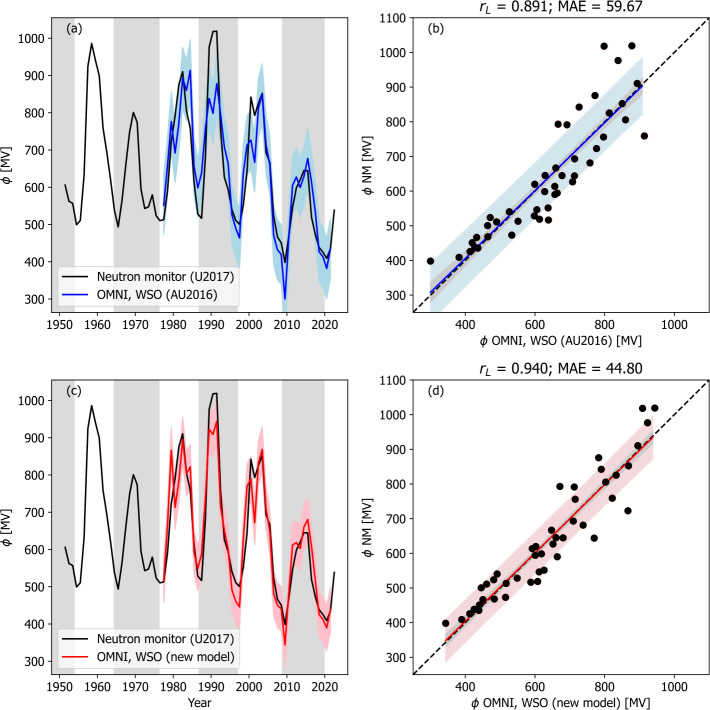
Models of $\phi $ applied to OMNI observations of $F_{S}$ and WSO observations of $\alpha $. The left-hand panels (a and c) show time series plots of $\phi $ derived neutron monitor data by U2017 (black) and from models (red or blue). Panels a and b show the original model of AU2016, and panels c and d show the modified model presented in this study. The coloured-shaded region shows the 1 $\sigma$ uncertainty range. The right-hand panels show the same data as a scatter plot. The black-dashed line is $y=x$. The coloured-shaded region shows the observed 1 $\sigma$ uncertainty range, whereas the grey-shaded region shows the 1 $\sigma$ uncertainty on the best fit line.

As we are using $\alpha $ based on WSO observations, $\phi $ can only be reconstructed over the interval from 1977 to 2021. Comparing with $\phi _{NM}$ over this interval, we find $r_{L} = 0.89$, as previously reported, and a mean absolute error (MAE) of 59.7 MV. As shown in Figure 4a, the AU2016 model driven with OMNI and WSO inputs is capturing the solar cycle trend, but the cycle-to-cycle trend appears to be underestimated. For example, the 1990 peak is the highest in the $\phi _{NM}$ record, but in the model reconstruction, it is slightly smaller than the 1980 peak.

We here modify the AU2016 model to produce a simpler combination of the same heliospheric parameters. As in AU2016, we assume that $\phi $ is proportional to ${F_{S}}^{n}$, with the HCS tilt and polarity as further multiplicative modifications. As with AU2016, this implicitly forces $\phi = 0$ when $F_{S} = 0$. In AU2016, $\alpha $ modified the power-law index between $\phi $ and $F_{S}$. We here instead assume that the HCS tilt modulation takes the form of $\sin \alpha $, as this approximates the volume of the heliosphere containing the HCS. Similarly, we assume that the polarity effect is determined by $p* = p (1-\sin \alpha *)$, rather than by $p$ itself, to account for the polarity being poorly defined when the $\alpha $ is high, such as times around the polarity reversal. Thus we use an expression of the form

4 $$ \phi _{new} = \phi _{0} {F_{S}} ^{n} (1 + A \sin \alpha ) (1 + B p*), $$

where $\phi _{0}$, $n$, $A$ and $B$ are free parameters to be determined by fitting to $\phi _{NM}$. These coefficients can be directly interpreted in terms of the strength of the HCS modulation and the polarity (and hence drift) modulation, respectively.

Fitting Equation 4 using OMNI $F_{S}$ and WSO $\alpha $ to $\phi _{NM}$, we obtain $\phi _{0} = 642$ MV, $n = 0.665$, $A = 0.488$ and $B = -0.0319$. As $\alpha $ varies between 6 and $70^{\circ}$, this suggests the HCS angle modulates $\phi $ by around 40%, whereas the polarity contribution is only around 6%.

The resulting model $\phi $ time series is shown in Figure 4c. The linear correlation is increased relative to AU2016 from $r_{L} = 0.891$ to 0.94, and the MAE reduced from 59.7 to 44.8 MV. However, the low number of data points ($N = 45$) means a Meng’s Z test finds that this difference is not statistically significant. But by eye we do note the cycle-to-cycle variation is stronger and in better agreement with $\phi _{NM}$, which is promising for long-term reconstructions and calibration of radionuclide estimates of $\phi $.

**Figure 5:**
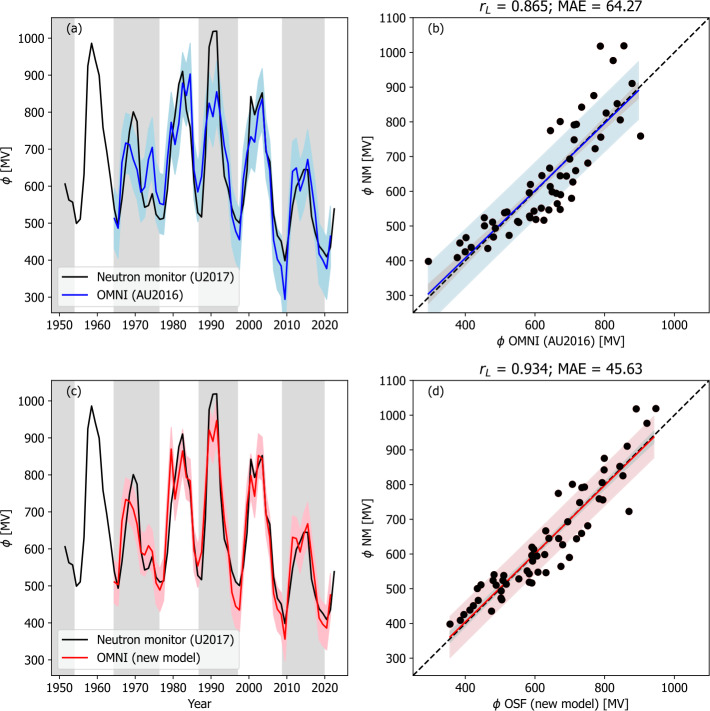
Models of $\phi $ applied to OMNI observations of $F_{S}$ and a cycle-averaged variation of $\alpha $. The left-hand panels (a and c) show time series plots of $\phi $ derived neutron monitor data by U2017 (black) and from models (red or blue). Panels a and b show the original model of AU2016, and panels c and d show the modified model presented in this study. The coloured-shaded region shows the 1 $\sigma$ uncertainty range. The right-hand panels show the same data as a scatter plot. The coloured line shows the best fit, the black-dashed line is $y=x$. The coloured-shaded region shows the 1 $\sigma$ uncertainty range, whereas the grey-shaded region shows the 1 $\sigma$ uncertainty on the best-fit line.

These model results use the observed $\alpha $ from WSO data. For historic reconstruction, it is necessary to reconstruct $\alpha $ on the basis of the solar cycle phase (Figure 3b). Figure 5 shows the two $\phi $ models applied to the OMNI $F_{S}$ and the solar-cycle averaged $\alpha $ variation. While the goodness of the fit drops slightly for both models compared with using the observed $\alpha $ (see Table 2), the decrease is not significant, suggesting this approach is valid. The additional 13 years of data ($N = 58$) means that a Meng’s Z test finds the improvement of the new model over AU2016 significant at the 95% confidence level.

## Modelling $\phi $ Using Reconstructions of $F_{S}$

**Figure 6:**
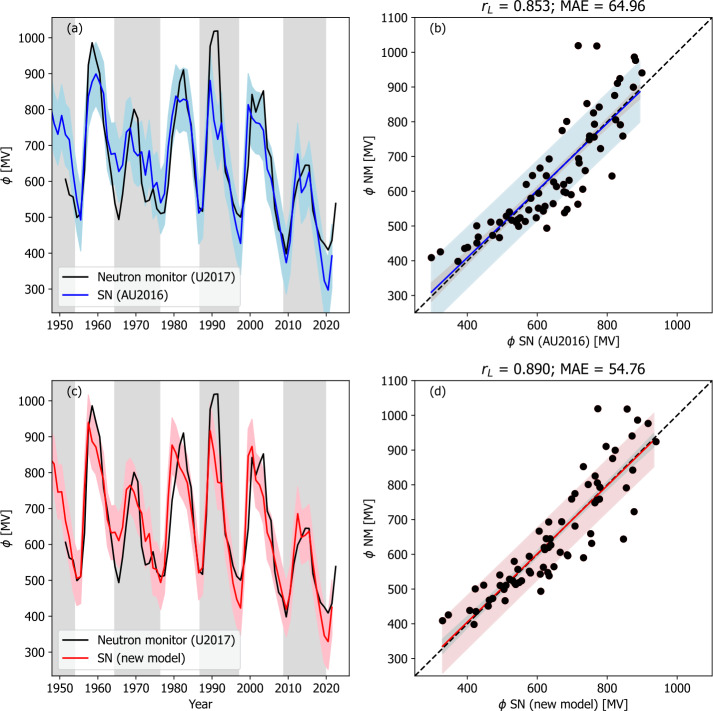
Models of $\phi $ applied to sunspot reconstructions of $F_{S}$ and a cycle-averaged variation of $\alpha $. The left-hand panels (a and c) show time series plots of $\phi $ derived neutron monitor data by U2017 (black) and from models (red or blue). Panels a and b show the original model of AU2016, and panels c and d show the modified model presented in this study. The coloured-shaded region shows the 1 $\sigma$ uncertainty range. The right-hand panels show the same data as a scatter plot. The coloured line shows the best fit, and the black-dashed line is $y=x$. The coloured-shaded region shows the 1 $\sigma$ uncertainty range, whereas the grey-shaded region shows the 1 $\sigma$ uncertainty on the best-fit line.

We now turn to the reconstructions of $F_{S}$ from proxy data. Figure 6 shows the model results using the SN-based reconstruction of $F_{S}$, with best-fit model parameters from Table 2. For both models, goodness-of-fit parameters are significantly lower than when using $F_{S}$ from OMNI data, as expected. By eye the cycle-to-cycle variation appears to be damped for both models. The inability to capture the long-term (i.e. cycle-to-cycle) variations potentially limits the use of SN-based reconstructions of $\phi $ for calibrating radionuclide estimates.

**Figure 7:**
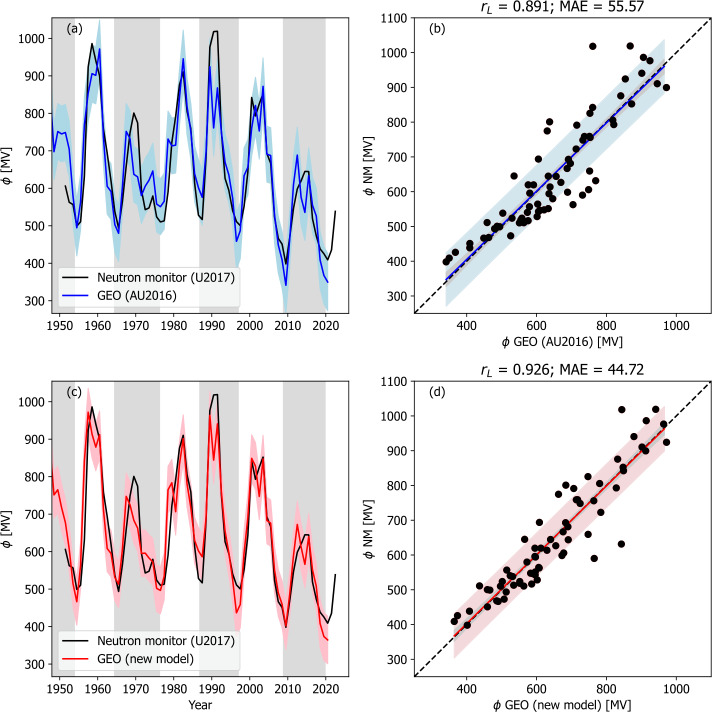
Models of $\phi $ applied to geomagnetic reconstructions of $F_{S}$ and a solar cycle-averaged profile of $\alpha $. The left-hand panels (a and c) show time series plots of $\phi $ derived neutron monitor data by U2017 (black) and from models (red or blue). Panels a and b show the original model of Asvestari and Usoskin (2016), and panels c and d show the modified model presented in this study. The coloured-shaded region shows the 1 $\sigma$ uncertainty range. The right-hand panels show the same data as a scatter plot. The coloured line shows the best fit, the black-dashed line is $y=x$. The coloured-shaded region shows the 1 $\sigma$ uncertainty range, whereas the grey-shaded region shows the 1 $\sigma$ uncertainty on the best-fit line.

Finally, the use of the models with the geomagnetic estimate of $\phi $ is shown in Figure 7. For both models, correlations are significantly higher than when using the SN-based $F_{S}$. Again, goodness of fit is higher for the new version of the model than for the original AU2016 form. We can see that the new model again captures more of the long-term variation in $\phi $ than the original AU2016 form and produces lower uncertainties. For these reasons, we use geomagnetic $F_{S}$ and the new model to extend $\phi $ back to 1845.

## Reconstructing $\phi $ over the Interval 1845 – Present

**Figure 8:**
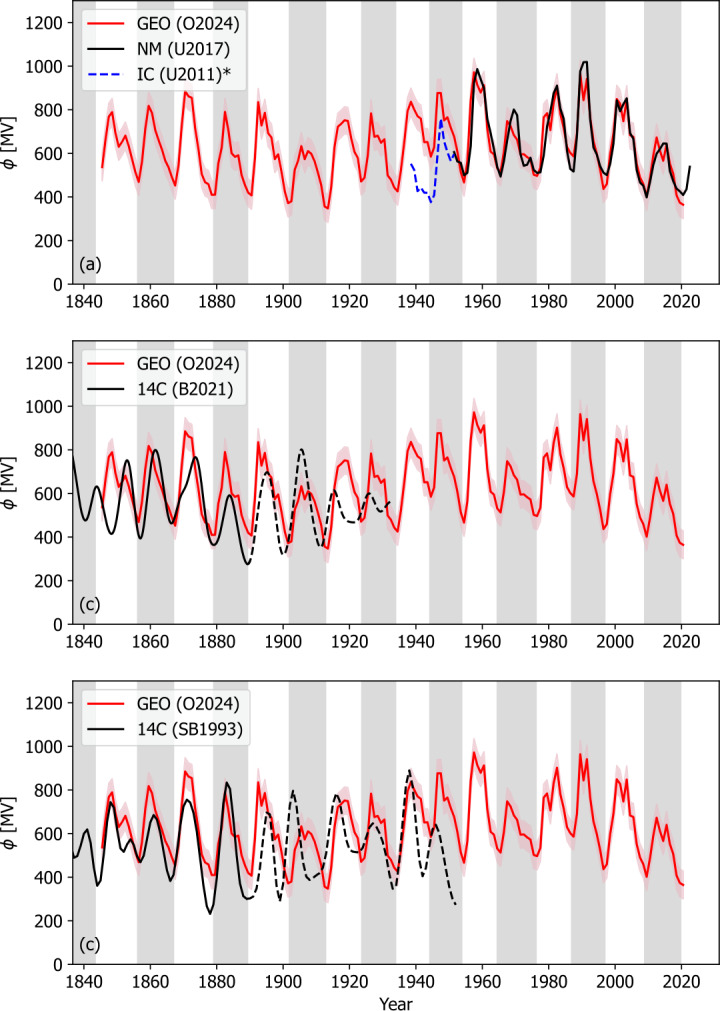
Estimates of $\phi$ over the interval 1840 – present. In all panels, the red line shows the $\phi$ reconstruction using the geomagnetic estimate of $F_{S}$ and the cycle-averaged estimate of $\alpha$ with shaded areas showing 1-sigma uncertainty bands. The black line in panel a shows the U2017. The blue dashed line shows the scaled ionisation chamber estimate of $\phi$. Panel b shows the radionuclide estimates of $\phi$, namely ^14^C-based estimate from Brehm et al. (2021). Panel c shows the equivalent Stuiver and Braziunas (1993) ^14^C. In both panels b and c, the dashed lines approximately indicate the influence of the Suess effect of burning fossil fuels.

Figure 8 shows the result of applying Equation 4 to the full geomagnetic $F_{S}$ reconstruction and the cycle-averaged $\alpha $ profile, based on solar minimum times defined by (Owens et al., 2024). The geomagnetic reconstruction of $\phi $ suggests that the two most recent solar minima – between Cycles 23 and 24 and Cycles 22 and 25 – have produced $\phi $ values $\approx 370 \pm 60$ MV, which is comparable to the lowest value in the 175-year reconstruction in 1913 of $\approx 355 \pm 70$ MV. The 1960 peak of $\phi \approx 975 \pm 50$ MV is the highest reconstructed value in the 175-year record. Thus the space age covers the full range of $\phi $ variability over the last 175 years.

Figure 8a shows the agreement between the reconstruction with $\phi _{NM}$ produced by U2017 and the scaled Usoskin, Bazilevskaya, and Kovaltsov (2011) ionisation chamber extension in blue. The variations from cycle to cycle agree well with the NM portion of the series. This is not the case for the ionisation chamber data, which shows different patterns of variation. This suggests that the ionisation chamber estimates of $\phi $ are not well calibrated with the NM record and should not be used to calibrate the radionuclide estimates of $\phi $.

Figures 8b and c shows the comparison with the $\phi$ estimates from ^14^C data from Brehm et al. (2021) and Stuiver and Braziunas (1993), respectively. There is approximately agreement in the overall level of heliospheric modulation.

## Summary and Discussion

In this study, we have produced the first estimate of the heliospheric modulation potential $\phi $ based on geomagnetic reconstructions of the open solar flux $F_{S}$. These are the most accurate $F_{S}$ on the centennial time scale. In this way, it is possible to provide a more accurate $\phi $ reconstruction than using sunspot estimates of $F_{S}$, but still extend the $\phi $ reconstruction back to 1845. This is approximately $\times 2.5$ the duration of the neutron monitor estimate of $\phi $ and provides significantly more overlap with the radionuclide records.

To provide this new geomagnetic estimate of $\phi $, we have made a number of updates and modifications to the model of Asvestari and Usoskin (2016) (AU2016):

- The cycle-averaged HCS tilt angle $\alpha $ profile was updated to use WSO data through 1977 to 2023. Rather than fitting a functional form to the average variation, we use a look-up table of the mean $\alpha $ for a given solar cycle phase.
- The relationship between $\phi $ and $F_{S}$ was modified to use a constant power-law index, rather than a varying index adjusted by $\alpha $.
- The relationship between $\phi $ and $\alpha $ is assumed to take the form of $\sin \alpha $, as this approximates the volume of the heliosphere occupied by the HCS.
- We define an effective polarity to account for the fact that the polarity is undefined when $\alpha = 90^{\circ}$. This removes a discontinuous behaviour of modelled $\phi $ at the time of the polarity reversal.

For the same input heliospheric parameters (namely $F_{S}$, $\alpha $, $p$) and the same number of free parameters, the new model is shown to provide a significantly improved agreement with $\phi _{NM}$ than AU2016. In particular, the long-term (cycle-to-cycle) variation is better reconstructed.

The new heliospheric parameter model is used to demonstrate:

- The best fit of the new heliospheric parameter model with $\phi _{NM}$ suggests that $\phi $ varies approximately as $F_{S} ^{0.65}$.
- The best fit also suggests $\alpha $ further modulates $\phi $ by around 25 to 45%.
- The best-fit parameters suggest that the heliospheric polarity only modulates $\phi $ by around 6 to 20%.
- Using a cycle-averaged profile for $\alpha $, rather than the directly observed time series, does not significantly affect the $\phi $ reconstruction.
- $\phi $ reconstructions based on geomagnetic $F_{S}$ show significantly better agreement with $\phi _{NM}$ than reconstructions based sunspot $F_{S}$.
- The ionisation chamber estimate of $\phi $ shows a significant deviation from the geomagnetic estimate. This is also true with ^10^Be data (Zheng et al., 2021).

Figures 8b and c show the comparison with current radionuclide estimates of $\phi$ . Given the disparate nature of the source data, there is reasonable correspondence in the overall modulation levels and relative variations over the solar cycles. We hope that future work can bring the radionuclide estimates into further agreement with this new geomagnetic benchmark. All data are provided as supplementary material.

## Supplementary Information

Below is the link to the electronic supplementary material.

(CSV 33 kB)

## Data Availability

Data is provided within the manuscript or supplementary information files

## Materials Availability

N/A

## Code Availability

The geomagnetic $\phi $ estimate is provided as Supplementary material. All code and supporting data can be accessed from https://github.com/University-of-Reading-Space-Science/GeomagneticPhi.

## References

[CR1] Acero, F.J., Gallego, M.C., García, J.A., Usoskin, I.G., Vaquero, J.M.: 2018, Extreme value theory applied to the millennial sunspot number series. *Astrophys. J.* **853**, 80. DOI.

[CR2] Alanko-Huotari, K., Usoskin, I.G., Mursula, K., Kovaltsov, G.A.: 2007, Cyclic variations of the heliospheric tilt angle and cosmic ray modulation. *Adv. Space Res.* **40**, 1064. DOI.

[CR3] Alken, P., Thébault, E., Beggan, C.D., Amit, H., Aubert, J., Baerenzung, J., Bondar, T.N., Brown, W.J., Califf, S., Chambodut, A., Chulliat, A., Cox, G.A., Finlay, C.C., Fournier, A., Gillet, N., Grayver, A., Hammer, M.D., Holschneider, M., Huder, L., Hulot, G., Jager, T., Kloss, C., Korte, M., Kuang, W., Kuvshinov, A., Langlais, B., Léger, J.-M., Lesur, V., Livermore, P.W., Lowes, F.J., Macmillan, S., Magnes, W., Mandea, M., Marsal, S., Matzka, J., Metman, M.C., Minami, T., Morschhauser, A., Mound, J.E., Nair, M., Nakano, S., Olsen, N., Pavón-Carrasco, F.J., Petrov, V.G., Ropp, G., Rother, M., Sabaka, T.J., Sanchez, S., Saturnino, D., Schnepf, N.R., Shen, X., Stolle, C., Tangborn, A., Tøffner-Clausen, L., Toh, H., Torta, J.M., Varner, J., Vervelidou, F., Vigneron, P., Wardinski, I., Wicht, J., Woods, A., Yang, Y., Zeren, Z., Zhou, B.: 2021, International geomagnetic reference field: the thirteenth generation. *Earth Planets Space* **73**, 49. DOI.

[CR4] Arlt, R.: 2011, The sunspot observations by Samuel Heinrich Schwabe. *Astron. Nachr.* **332**, 805. DOI.

[CR5] Asvestari, E., Usoskin, I.G.: 2016, An empirical model of heliospheric cosmic ray modulation on long-term time scale. *J. Space Weather Space Clim.* **6**, A15. DOI.

[CR6] Baxter, M.S., Walton, A.: 1970, A theoretical approach to the suess effect. *Proc. Roy. Soc. A* **318**, 213.

[CR7] Beer, J., Blinov, A., Bonani, G., Finkel, R.C., Hofmann, H.J., Lehmann, B., Oeschger, H., Sigg, A., Schwander, J., Staffelbach, T., Stauffer, B., Suter, M., Wötfli, W.: 1990, Use of 10Be in polar ice to trace the 11-year cycle of solar activity. *Nature* **347**, 164. DOI.

[CR8] Beer, J., McCracken, K.G., Abreu, J., Heikkilä, U., Steinhilber, F.: 2013, Cosmogenic radionuclides as an extension of the neutron monitor era into the past: potential and limitations. *Space Sci. Rev.* **176**, 89. DOI.

[CR9] Berggren, A.-M., Beer, J., Possnert, G., Aldahan, A., Kubik, P., Christl, M., Johnsen, S.J., Abreu, J., Vinther, B.M.: 2009, A 600-year annual 10Be record from the NGRIP ice core, Greenland. *Geophys. Res. Lett.* 36. DOI.

[CR10] Brehm, N., Bayliss, A., Christl, M., Synal, H.-A., Adolphi, F., Beer, J., Kromer, B., Muscheler, R., Solanki, S.K., Usoskin, I., Bleicher, N., Bollhalder, S., Tyers, C., Wacker, L.: 2021, Eleven-year solar cycles over the last millennium revealed by radiocarbon in tree rings. *Nat. Geosci.* **14**, 10. DOI.

[CR11] Caballero-Lopez, R.A., Engelbrecht, N.E., Richardson, J.D.: 2019, Correlation of long-term cosmic-ray modulation with solar activity parameters. *Astrophys. J.* **883**, 73. DOI.

[CR12] Clette, F., Lefèvre, L.: 2016, The new sunspot number: assembling all corrections. *Solar Phys.* **291**, 2629. DOI.

[CR13] Clette, F., Lefèvre, L., Chatzistergos, T., Hayakawa, H., Carrasco, V.M.S., Arlt, R., Cliver, E.W., Dudok de Wit, T., Friedli, T.K., Karachik, N., Kopp, G., Lockwood, M., Mathieu, S., Muñoz-Jaramillo, A., Owens, M., Pesnell, D., Pevtsov, A., Svalgaard, L., Usoskin, I.G., van Driel-Gesztelyi, L., Vaquero, J.M.: 2023, Recalibration of the sunspot-number: status report. *Solar Phys.* **298**, 44. DOI.

[CR14] Cliver, E.W., Ling, A.G.: 2001, 22 year patterns in the relationship of sunspot number and tilt angle to cosmic-ray intensity. *Astrophys. J. Lett.* **551**, L189. DOI.

[CR15] Frost, A.M., Owens, M., Macneil, A., Lockwood, M.: 2022, Estimating the open solar flux from in-situ measurements. *Solar Phys.* **297**, 82. DOI.

[CR16] Gleeson, L.J., Axford, W.I.: 1968, Solar modulation of galactic cosmic rays. *Astrophys. J.* **154**, 1011. DOI.

[CR17] Golubenko, K., Rozanov, E., Kovaltsov, G., Usoskin, I.: 2022, Zonal mean distribution of cosmogenic isotope (^7^Be, ^10^Be, ^14^C, and ^36^Cl) production in stratosphere and troposphere. *J. Geophys. Res.* **127**, e2022JD036726. DOI.

[CR18] Hathaway, D.H.: 2010, The solar cycle. *Living Rev. Solar Phys.* **7**, 1. DOI. 10.1007/lrsp-2015-4 PMC4841188 27194958

[CR19] Hayakawa, H., Hattori, K., Sôma, M., Iju, T., Besser, B.P., Kosaka, S.: 2022, An overview of sunspot observations in 1727 – 1748. *Astrophys. J.* **941**, 151. DOI.

[CR20] Herbst, K., Muscheler, R., Heber, B.: 2017, The new local interstellar spectra and their influence on the production rates of the cosmogenic radionuclides 10Be and 14C. *J. Geophys. Res.* **122**, 23. DOI.

[CR21] Herbst, K., Kopp, A., Heber, B., Steinhilber, F., Fichtner, H., Scherer, K., Matthiä, D.: 2010, On the importance of the local interstellar spectrum for the solar modulation parameter. *J. Geophys. Res.* 115. DOI.

[CR22] Hoeksema, J.T.: 1991, Large-scale solar and heliospheric magnetic fields. *Adv. Space Res.* **11**, 15.

[CR23] Jokipii, J.R., Levy, E.H., Hubbard, W.B.: 1977, Effects of particle drift on cosmic-ray transport. I - General properties, application to solar modulation. *Astrophys. J.* **213**, 861. DOI.

[CR24] Kilpua, E., Koskinen, H.E.J., Pulkkinen, T.I.: 2017, Coronal mass ejections and their sheath regions in interplanetary space. *Living Rev. Solar Phys.* **14**, 5. DOI. 10.1007/s41116-017-0009-6 PMC6956910 31997985

[CR25] King, J.H., Papitashvili, N.E.: 2005, Solar Wind Spatial Scales in and Comparisons of Hourly Wind and ACE Plasma and Magnetic Field Data. *J. Geophys. Res.* 110. DOI.

[CR26] Kovaltsov, G.A., Mishev, A., Usoskin, I.G.: 2012, A new model of cosmogenic production of radiocarbon 14C in the atmosphere. *Earth Planet. Sci. Lett.* **337**, 114. DOI.

[CR27] Kovaltsov, G.A., Usoskin, I.G.: 2010, A new 3D numerical model of cosmogenic nuclide 10Be production in the atmosphere. *Earth Planet. Sci. Lett.* **291**, 182. DOI.

[CR28] Krivova, N.A., Solanki, S.K., Hofer, B., Wu, C.-J., Usoskin, I.G., Cameron, R.: 2021, Modelling the evolution of the Sun’s open and total magnetic flux. *Astron. Astrophys.* **650**, A70. DOI.

[CR29] Lal, D., Peters, B.: 1967, Cosmic ray produced radioactivity on the Earth. In: Sitte, K. (ed.) *Kosmische Strahlung II / Cosmic Rays II* **551**, Springer, Berlin, 978. DOI.

[CR30] Li, J.-T., Beacom, J.F., Peter, A.H.G.: 2022, Galactic cosmic-ray propagation in the inner heliosphere: improved force-field model. *Astrophys. J.* **937**, 27. DOI.

[CR31] Lockwood, M., Owens, M.J.: 2014, Centennial variations in sunspot number, open solar flux and streamer belt width: 3. Modeling. *J. Geophys. Res.* **119**, 5193. DOI.

[CR32] Lockwood, M., Owens, M.: 2024, Reconstruction of Carrington rotation means of open solar flux over the past 154 years. *Solar Phys.* **299**, 28. DOI.

[CR33] Lockwood, M., Owens, M.J., Barnard, L.: 2014, Centennial variations in sunspot number, open solar flux, and streamer belt width: 1. Correction of the sunspot number record since 1874. *J. Geophys. Res.* **119**, 5172. DOI.

[CR34] Lockwood, M., Owens, M.J., Barnard, L.A., Scott, C.J., Frost, A.M., Yu, B., Chi, Y.: 2022, Application of historic datasets to understanding open solar flux and the 20th-century grand solar maximum. 1. Geomagnetic, ionospheric, and sunspot observations. *Front. Astron. Space Sci.* 9. DOI.

[CR35] McCracken, K.G., Beer, J.: 2007, Long-term changes in the cosmic ray intensity at Earth, 1428 – 2005. *J. Geophys. Res.* 112. DOI.

[CR36] Moloto, K.D., Engelbrecht, N.E.: 2020, A fully time-dependent ab initio cosmic-ray modulation model applied to historical cosmic-ray modulation. *Astrophys. J.* **894**, 121. DOI.

[CR37] Moraal, H.: 2013, Cosmic-ray modulation equations. *Space Sci. Rev.* **176**, 299. DOI.

[CR38] Muscheler, R., Joos, F., Beer, J., Müller, S.A., Vonmoos, M., Snowball, I.: 2007, Solar activity during the last 1000yr inferred from radionuclide records. *Quat. Sci. Rev.* **26**, 82. DOI.

[CR39] Muscheler, R., Adolphi, F., Herbst, K., Nilsson, A.: 2016, The revised sunspot record in comparison to cosmogenic radionuclide-based solar activity reconstructions. *Solar Phys.* **291**, 3025. DOI.

[CR40] Nguyen, H.L.: 2023, Solar variability over the Holocene period: disentangling geomagnetic and solar influences on a new continuous 10Be record from Little Dome C, Antarctica. Doctoral Thesis (compilation), Lund. ISBN 9789187847745.

[CR41] Owens, M.: 2020, Solar-wind structure. *Oxf. Res. Encycl. Phys.* DOI.

[CR42] Owens, M.J., Crooker, N.U., Lockwood, M.: 2011, How is open solar magnetic flux lost over the solar cycle? *J. Geophys. Res.* **116**, A04111. DOI.

[CR43] Owens, M.J., Forsyth, R.J.: 2013, The heliospheric magnetic field. *Living Rev. Solar Phys.* **10**, 5. DOI.

[CR44] Owens, M.J., Lockwood, M.: 2012, Cyclic loss of open solar flux since 1868: the link to heliospheric current sheet tilt and implications for the Maunder minimum. *J. Geophys. Res.* **117**, A04102. DOI.

[CR45] Owens, M.J., Lockwood, M., Barnard, L., Davis, C.J.: 2011, Solar cycle 24: implications for energetic particles and long-term space climate change. *Geophys. Res. Lett.* **38**, 1. DOI.

[CR46] Owens, M.J., Cliver, E.W., McCracken, K.G., Beer, J., Balogh, A., Barnard, L., Lockwood, M., Rouillard, A.P., Wang, Y.-M., Passos, S., Riley, P., Usoskin, I.: 2016, Near-Earth heliospheric magnetic field intensity since 1750. Part 2: cosmogenic radionuclide reconstructions. *J. Geophys. Res.* **121**, 6064. DOI.

[CR47] Owens, M.J., Lockwood, M., Riley, P., Linker, J.: 2017, Sunward strahl: a method to unambiguously determine open solar flux from in situ spacecraft measurements using suprathermal electron data. *J. Geophys. Res.* **122**, 910. DOI.

[CR48] Owens, M.J., Lockwood, M., Barnard, L.A., Scott, C.J., Haines, C., Macneil, A.: 2021, Extreme space-weather events and the solar cycle. *Solar Phys.* **296**, 82. DOI.

[CR49] Owens, M.J., Lockwood, M., Barnard, L.A., Usoskin, I., Hayakawa, H., Pope, B.J.S., McCracken, K.: 2024, Reconstructing sunspot number by forward-modelling open solar flux. *Solar Phys.* **299**, 3. DOI.

[CR50] Paleari, C.I., Mekhaldi, F., Erhardt, T., Zheng, M., Christl, M., Adolphi, F., Hörhold, M., Muscheler, R.: 2023, Evaluating the 11-year solar cycle and short-term ^10^Be deposition events with novel excess water samples from the East Greenland Ice-core Project (EGRIP). *Clim. Past* **19**, 2409. DOI.

[CR51] Parker, E.N.: 1958, Dynamics of the interplanetary gas and magnetic fields. *Astrophys. J.* **128**, 664. DOI.

[CR52] Parker, E.N.: 1965, The passage of energetic charged particles through interplanetary space. *Planet. Space Sci.* **13**, 9. DOI.

[CR53] Pavón-Carrasco, F.J., Gómez-Paccard, M., Campuzano, S.A., González-Rouco, J.F., Osete, M.L.: 2018, Multi-centennial fluctuations of radionuclide production rates are modulated by the Earth’s magnetic field. *Sci. Rep.* **8**, 9820. DOI. 29959376 10.1038/s41598-018-28115-4 PMC6026124

[CR54] Potgieter, M.: 2013, Solar modulation of cosmic rays. *Living Rev. Solar Phys.* **10**, 3. DOI.

[CR55] Roth, R., Joos, F.: 2013, A reconstruction of radiocarbon production and total solar irradiance from the Holocene 14 C and CO 2 records: implications of data and model uncertainties. *Clim. Past* **9**, 1879. DOI.

[CR56] Smith, E.J.: 1990, The heliospheric current sheet and modulation of Galactic cosmic rays. *J. Geophys. Res.* **95**, 18731. DOI.

[CR57] Snowball, I., Muscheler, R.: 2007, Palaeomagnetic intensity data: an Achilles heel of solar activity reconstructions. *Holocene* **17**, 851. DOI.

[CR58] Stuiver, M., Braziunas, T.F.: 1993, Sun, ocean, climate and atmospheric 14CO2: an evaluation of causal and spectral relationships. *Holocene* **3**, 289. DOI.

[CR59] Stuiver, M., Quay, P.D.: 1980, Patterns of atmospheric 14C changes. *Radiocarbon* **22**, 166. DOI.

[CR60] Thomas, S.R., Owens, M.J., Lockwood, M.: 2013, The 22-year Hale cycle in cosmic ray flux – evidence for direct heliospheric modulation. *Solar Phys.* **289**, 407. DOI.

[CR61] Usoskin, I.G.: 2017, A History of Solar Activity over Millennia. *Living Rev. Solar Phys.* 14. DOI.

[CR62] Usoskin, I.G.: 2023, A history of solar activity over millennia. *Living Rev. Solar Phys.* **20**, 2. DOI.

[CR63] Usoskin, I.G., Bazilevskaya, G.A., Kovaltsov, G.A.: 2011, Solar modulation parameter for cosmic rays since 1936 reconstructed from ground-based neutron monitors and ionization chambers. *J. Geophys. Res.* 116. DOI.

[CR64] Usoskin, I.G., Alanko-Huotari, K., Kovaltsov, G.A., Mursula, K.: 2005, Heliospheric modulation of cosmic rays: Monthly reconstruction for 1951 – 2004. *J. Geophys. Res.* 110. DOI.

[CR65] Usoskin, I., Arlt, R., Asvestari, E., Hawkins, E., Kapyla, M., Kovaltsov, G.A., Krivova, N.A., Lockwood, M., Mursula, K., O’Reilly, J., Owens, M.J., Scott, C., Sokoloff, D.D., Solanki, S.K., Soon, W., Vaquero, J.M.: 2015, The Maunder minimum (1645 – 1715) was indeed a grand minimum: a reassessment of multiple datasets. *Astron. Astrophys.* **581**, A95. DOI.

[CR66] Usoskin, I.G., Gil, A., Kovaltsov, G.A., Mishev, A.L., Mikhailov, V.V.: 2017, Heliospheric modulation of cosmic rays during the neutron monitor era: calibration using PAMELA data for 2006 – 2010. *J. Geophys. Res.* **122**, 3875. DOI.

[CR67] Usoskin, I.G., Solanki, S.K., Krivova, N.A., Hofer, B., Kovaltsov, G.A., Wacker, L., Brehm, N., Kromer, B.: 2021, Solar cyclic activity over the last millennium reconstructed from annual 14C data. *Astron. Astrophys.* **649**, A141. DOI.

[CR68] Väisänen, P., Usoskin, I., Mursula, K.: 2021, Seven decades of neutron monitors (1951 – 2019): overview and evaluation of data sources. *J. Geophys. Res.* **126**, e28941. DOI.

[CR69] Väisänen, P., Usoskin, I., Kähkönen, R., Koldobskiy, S., Mursula, K.: 2023, Revised reconstruction of the heliospheric modulation potential for 1964 – 2022. *J. Geophys. Res.* **128**, e2023JA031352. DOI.

[CR70] Vaquero, J.M., Svalgaard, L., Carrasco, V.M.S., Clette, F., Lefèvre, L., Gallego, M.C., Arlt, R., Aparicio, A.J.P., Richard, J., Howe, R.: 2016, A revised collection of sunspot group numbers. *Solar Phys.* **291**, 3061. DOI.

[CR71] Vennerstrom, S., Lefevre, L., Dumbovic, M., Crosby, N., Malandraki, O., Patsou, I., Clette, F., Veronig, A., Vrsnak, B., Leer, K., Moretto, T.: 2016, Extreme geomagnetic storms - 1868 – 2010. *Solar Phys.* **291**, 1447. DOI.

[CR72] Vonmoos, M., Beer, J., Muscheler, R.: 2006, Large variations in Holocene solar activity: Constraints from 10Be in the Greenland Ice Core Project ice core. *J. Geophys. Res.* 111. DOI.

[CR73] Vos, E.E., Potgieter, M.S.: 2015, New modeling of galactic proton modulation during the minium of solar cycle 23/24. *Astrophys. J.* **815**, 119. DOI.

[CR74] Zheng, M., Adolphi, F., Sjolte, J., Aldahan, A., Possnert, G., Wu, M., Chen, P., Muscheler, R.: 2021, Solar activity of the past 100 years inferred from 10Be in ice cores—implications for long-term solar activity reconstructions. *Geophys. Res. Lett.* **48**, e2020GL090896. DOI.

[CR75] Zheng, M., Adolphi, F., Paleari, C., Tao, Q., Erhardt, T., Christl, M., Wu, M., Lu, Z., Hörhold, M., Chen, P., Muscheler, R.: 2023, Solar, atmospheric, and volcanic impacts on 10Be depositions in Greenland and Antarctica during the last 100 years. *J. Geophys. Res.* **128**, e2022JD038392. DOI.

